# Human Rhinovirus Induced Cytokine/Chemokine Responses in Human Airway Epithelial and Immune Cells

**DOI:** 10.1371/journal.pone.0114322

**Published:** 2014-12-12

**Authors:** Devi Rajan, Courtney E. McCracken, Hannah B. Kopleman, Shuya Y. Kyu, F. Eun-Hyung Lee, Xiaoyan Lu, Larry J. Anderson

**Affiliations:** 1 Department of Pediatrics, Emory Children's Center, Atlanta, Georgia, United States of America; 2 Division of Pulmonary, Allergy, & Critical Care Medicine, Emory University, Atlanta, Georgia, United States of America; 3 Division of Viral Diseases, Centers for Disease Control and Prevention, Atlanta, Georgia, United States of America; University of San Francisco, United States of America

## Abstract

Infections with human rhinovirus (HRV) are commonly associated with acute upper and lower respiratory tract disease and asthma exacerbations. The role that HRVs play in these diseases suggests it is important to understand host-specific or virus-specific factors that contribute to pathogenesis. Since species A HRVs are often associated with more serious HRV disease than species B HRVs, differences in immune responses they induce should inform disease pathogenesis. To identify species differences in induced responses, we evaluated 3 species A viruses, HRV 25, 31 and 36 and 3 species B viruses, HRV 4, 35 and 48 by exposing human PBMCs to HRV infected Calu-3 cells. To evaluate the potential effect of memory induced by previous HRV infection on study responses, we tested cord blood mononuclear cells that should be HRV naïve. There were HRV-associated increases (significant increase compared to mock-infected cells) for one or more HRVs for IP-10 and IL-15 that was unaffected by addition of PBMCs, for MIP-1α, MIP-1β, IFN-α, and HGF only with addition of PBMCs, and for ENA-78 only without addition of PBMCs. All three species B HRVs induced higher levels, compared to A HRVs, of MIP-1α and MIP-1β with PBMCs and ENA-78 without PBMCs. In contrast, addition of CBMCs had less effect and did not induce MIP-1α, MIP-1β, or IFN-α nor block ENA-78 production. Addition of CBMCs did, however, increase IP-10 levels for HRV 35 and HRV 36 infection. The presence of an effect with PBMCs and no effect with CBMCs for some responses suggest differences between the two types of cells possibly because of the presence of HRV memory responses in PBMCs and not CBMCs or limited response capacity for the immature CBMCs relative to PBMCs. Thus, our results indicate that different HRV strains can induce different patterns of cytokines and chemokines; some of these differences may be due to differences in memory responses induced by past HRV infections, and other differences related to virus factors that can inform disease pathogenesis.

## Introduction

Human rhinovirus (HRV) is a positive, single stranded RNA virus which belongs to the genus *Enterovirus* in the family *Picornaviridae*. More than 100 genotypes of HRVs have been identified that are phylogenetically distinct and divisible into three species, A, B, and C. Ninety percent of species A and B viruses use inter-cellular adhesion molecule 1 (ICAM1) or CD54 as their receptor [1], [2], whereas the others use low-density lipoprotein receptor (LDLR). The receptor for species C HRVs has not yet been identified but is considered to be distinct from those for species A and B HRVs [3]. HRVs are a primary cause of the common cold and are frequently associated with exacerbations of asthma and also cause lower respiratory tract disease including bronchitis, bronchiolitis and pneumonia [4], [5], [6], [7], [8]. HRV infection of cells triggers cytokine and chemokine production that may contribute to disease [9], [10], [11], [12], [13], [14], [15], [16]. Since epidemiologic studies suggest that species B virus is less commonly and species A and C HRVs are more frequently associated with disease [17], [18], [19], [20], [21], [22], [23], [24], we wondered if species differences in disease may be accompanied by differences in induction of cytokines or chemokines.

Several groups have noted differences among HRV serotypes in their *in vitro* response to infection. Wark and colleagues [25] measured release of IL-6, IP-10, IFN-β and IFN-γ by HRV-infected primary bronchial epithelial cells and noted higher levels induced by recent clinical isolates compared to laboratory passaged strains and differences in levels between cells infected with HRVs from the major and minor receptor groups. They also noted differences in levels produced by infected primary bronchial epithelial cells from asthmatic compared to non-asthmatic patients. We recently reported [26] differences between HRV 14 and HRV 16 in up or down regulation of several cytokines including those that are linked to airway inflammation. In these studies, we used a two-chamber trans-well tissue culture system with a human airway epithelial cell (HAEC) line derived from an adenocarcinoma of the lung, calu-3 cells, in the lower chamber and human peripheral blood mononuclear cells (PBMCs) in the upper apical chamber [26]. This system provides a model of the human local response to HRV infection and a way to study the effect of differences in HAECs, HRVs or PBMCs on the response to infection. With this system, we noted differences in cytokines and chemokines, like FGF-Basic, IL-15, IL-6, IL-28A, ENA-78 and IP-10 without PBMCs and MIP-1β, IL-28A, MCP-2, and IFN-α with PBMCs between HRV 14 and 16 during infection of calu-3 cells [26].

Since HRV 14 is a species B and HRV 16 a species A virus and both utilize ICAM-1 receptor, we hypothesized that some of the differences in cytokines and chemokines we noted might be associated with one or the other species. Hence to look into this possibility, we investigated 3 other species A HRV 25, 31 and 36 and 3 other species B viruses HRV 4, 35 and 48 using the two-chamber tissue culture system. These HRVs are among the many detected in recent studies of acute respiratory disease, replicate in our tissue culture system, include HRVs using both receptors, and some, HRV 14, 16, and 48, have been previously described in studies of the HRV immune response [26], [27], [28], [29], In addition, we also investigated the response of cord blood mononuclear cells (CBMCs) to HRV infection and compared these responses to those by adult PBMCs. CBMCs should be HRV naïve and, thus, eliminate memory responses that might confound results with PBMCs.

## Methods

### Ethics statement

Collection of blood for PBMC and CBMC studies was done with written informed consent and under an Emory University Institutional Review Board approved protocol (IRB #-00045690 and IRB # 60341) and University of Rochester approved protocol (# 21058).

### PBMCs and CBMCs

Mononuclear cells from the blood of 3 different adult donors and from cord blood of 3 different infants were purified using ficoll-histopaque density gradient centrifugation. Briefly, the blood or cord blood was diluted with 2 parts PBS to 1 part blood, layered over Lymphocyte Separation Medium (Cellgro), centrifuged at 800xg for 20min, the buffy coat layer was collected and washed twice in RPMI, and the mononuclear cells counted, divided into aliquots, and stored in liquid nitrogen until use. We chose to use cryopreserved cells for this study because they provide a consistent source of cells and have often been used for these types of studies.

### Human Airway Epithelial Cells (HAEC) and Viruses

Calu-3 cells were obtained from American Type Culture Collection (ATCC) and grown in minimum essential medium (MEM) supplemented with 10% fetal calf serum (FCS), 1mM L- Glutamine, 1mM Hepes and 1X non-essential amino acids (calu-3 media) and incubated at 37°C under 5% CO_2_. Three species A, HRV 25 (A), HRV 31 (A), and HRV 36 (A) and three species B, HRV 4 (B), HRV 35 (B) and HRV 48 (B), prototype strains were provided by the Centers for Disease Control and Prevention (Dr. Dean Erdman). All viruses have been detected in recent community studies of HRV [22], [27], grow well in our Calu-3/PBMC tissue culture system and include HRVs representing both receptors groups, i.e. all species B study viruses and the species A HRV 36 use ICAM1 and the species A HRV 25 and 31 uses LDLR. HRV 14, 16 and 48 have been described in earlier studies of HRV response in human AECs [26], [27], [28], [29]. The viruses were grown in HeLa cells maintained in MEM containing 10% FCS, 1% penicillin streptomycin and 1% L-Glutamine at 35°C under 5% CO_2_. HRV stocks were prepared by infecting monolayers of HeLa cells. The infected cells were maintained at 35°C under 5% CO_2_ until cytopathic effect (CPE) exceeded 70%. The media was collected and centrifuged briefly to remove the cellular debris. Virus was purified from the resultant supernatant, by centrifugation through a 20% sucrose cushion gradient at 20,000xg for 2 hours and re-suspending the pellet in MEM. The purified virus was divided into aliquots and stored at −80°C.

To determine the virus titer, Hela cells were grown in 96 well flat bottom tissue culture plates (5000 cells/well) and infected with 10-fold serial dilutions of virus in 8 replicates. The infected cells were incubated for 5 days and wells evaluated daily for CPE by microscopic examination. The tissue culture infectivity dose (TCID_50_) of the viruses was calculated based on the 5 day CPE using the Reed Muench method. Calu-3 cells seeded in 24 well plates were infected with 0.05 MOI of different viruses and supernatants from virus infected wells and control wells were collected daily to assess levels of viral RNA. The collected media were briefly centrifuged to remove the cell debris and RNA was extracted using Qiagen RNeasy mini kit according to manufacturer's instructions. HRV RNA was assayed by a real-time RT-PCR assay using One-Step RT-PCR Reagents (Life Technologies) and the Applied Biosystems 7500 Fast Real-Time PCR System (Life Technologies Corporation, Carlsbad, CA) as previously described [30].

### HAEC and PBMC co-culture

For the co-culture studies, calu-3 cells were seeded and infected with 0.05 MOI of infectious virus and mock-infected cell control material in duplicate wells. The inoculum was removed 2 hours later and 500 µl of calu-3 media was added to each well. The media was removed and fresh media was added one day post infection (p.i.). One day later, i.e. 2 days p.i., trans-well inserts (polyester trans-well inserts, 0.33 cm with 0.4 u pores, Corning, Inc., Corning, NY) with one million live PBMCs (viability determined by trypan blue exclusion) were placed in the virus infected and control wells. Media was collected from the basolateral chamber at 6, 24, and 48 hours after addition of the PBMCs. The collected media was centrifuged, stored at −80°C, and later tested by multiplex luminex assays according to the manufacturer's instructions for FGF-Basic, IFN-γ, IL–12 (p40 / p70), IL–13, RANTES, MIP–1α, MIG, MIP–1β, VEGF, IL–1β, IL–2, IL–4, IL–5, IL–6, IL–2R, MCP–1, Eotaxin, IL–8, IL–10, IL–15, IL–17, IL–1RA, GM–CSF, G–CSF, EGF, HGF, TNF-α, IL–7, IP-10, IFN-α (Human cytokine 30-plex panel, Life technologies) and ENA-78, MCP-2 and IL-28A (Human cytokine 3 plex panel, Millipore).

### Statistical Analyses

Statistical analyses were conducted using SAS 9.2 (Cary, NC) and statistical significance was assessed at P<0.05 unless otherwise noted. Due to the small sample sizes and non-normal data, nonparametric procedures were utilized. Specifically, for each cytokine, the actual data values were replaced by their rank in the dataset (i.e., their relative position in the dataset after ordering from least to greatest) and the analyses were carried out on the ranks of the data. The median and range were used to summarize cytokine levels. Prior to any modeling, the Wilcoxen rank sum test was used to identify cytokines that had HRV-associated responses to one or more HRVs, i.e. levels with HRV-infection were significantly different from levels with mock-infected control. Cytokines with HRV-associated responses were then included in the final analyses. Two-factor analysis of variance models were used to assess the effect of PBMC (yes/no) and species type (A/B/Control). All models initially included an interaction between PBMC and species type, but was removed from the model if it was not significant. For models with a significant interaction, we compared the effect of species on cytokine expression with and without PBMCs, and the effect of PBMCs on cytokine expression for each species. For models with a significant interaction, 3 sets of comparisons were made. The first two sets of comparisons were made among species with and without PBMCs using 3 planned pairwise comparisons: (1) A vs. Control, (2) B vs. Control, and (3) A vs. B. The third set of pairwise comparisons examined the effect of PBMC within each species using 3 additional pairwise comparisons: (1) A-*no PBMC* vs. A- *with PBMC*, (2) B-*no PBMC* vs. B- *with PBMC* and (3) Control-*no PBMC* vs. Control- *with PBMC.* To control for multiple comparisons, a Bonferroni adjustment was used in each set of pairwise comparisons and statistical significance was assessed using a significance level of 0.05/3 = 0.017.

## Results

As we and others have noted, different HRVs induce different patterns of secreted cytokines and chemokines (Table 1). Most of the HRV-associated responses (significantly different from mock-infected cells, P<0.05 by Wilcoxen rank sum test) were evident at 24 hours post PBMC addition i.e., 3 days p.i. HRV-associated response were detected for IFN-α, MIP-1α, MIP-1β, epithelial neutrophil activation protein-78 (ENA-78), IP-10, IL-15 and HGF (Table 1). There was variation in the pattern of responses among the HRVs. For example, only HRV 48, a species B virus, induced a significant increase in IP-10 (P<0.05) and only the 3 species B HRVs showed increases in MIP-1α, MIP-1β and ENA-78 (Table 1). As we expected, the addition of PBMCs to the infected cells affected results for some but not all cytokines. For example, the addition of PBMCs to all HRV-infected cells induced higher levels of IFN-α, HGF and MCP-1 with the exception of HRV 31 for HGF and MCP-1 (Table 1). The increases for IFN-α and HGF resulted in significant differences (P<0.05) compared to controls but not so for MCP-1. The addition of PBMCs resulted in a substantial increase in levels of MCP-1 for both HRV infected and mock infected cells resulting in no HRV-associated increase. There was a suggestion of an increase in MCP-1 levels after addition of PBMCs for individual species B HRVs (P = 0.051 to 0.084)) but did not achieve significance. The change in cytokine or chemokine levels after the addition of PBMCs for a given HRV was comparable for all 3 donor PBMCs.

**Table 1:** HRV-associated responses in calu-3 cells with or without PBMCs.

PBMC	Virus	MIP-1α	MIP-1β	MCP-1	ENA-78	IP-10	IFN-α	HGF	IL-15
**No**	**HRV 25**	58	42	376	20840	155	43	70	217*
	**HRV 31**	58	47	321	20230	75*	45	51	163
	**HRV 36**	66	53	333	21868	131	50	59	215
	**HRV 4**	50	42	288	43487*	103	38	52	147
	**HRV 35**	67	42	348	62448*	119	24	44	181*
	**HRV 48**	58	51	266	46628*	500*	54	122	205*
	**Control**	66	49	408	24423	140	43	21	95
**Yes**	**HRV 25**	76	116	10697	29776	127*	229*	158*	216*
	**HRV 31**	62	70	8978	23703	67	227*	113	147
	**HRV 36**	55	84	18180	20000*	64	209*	126*	166
	**HRV 4**	181*	254*	22378	29705	53	222*	177*	181
	**HRV 35**	184*	254*	18614	28132	76	242*	132*	144
	**HRV 48**	255*	340*	19696	23049	434*	245*	186*	181
	**Control**	69	83	8572	29968	86	62	43	109

Shown are the median values of cytokines and chemokines (pg/ml) produced in response to various HRVs and uninfected control infections at 24 hours after the time inserts with PBMCs were added (n = 3). Significant differences in cytokine/chemokine levels for HRV infected cells when compared to uninfected controls were calculated using Wilcoxen rank sum tests. * indicates significantly different from control (p<0.05).

**Figure 1:**
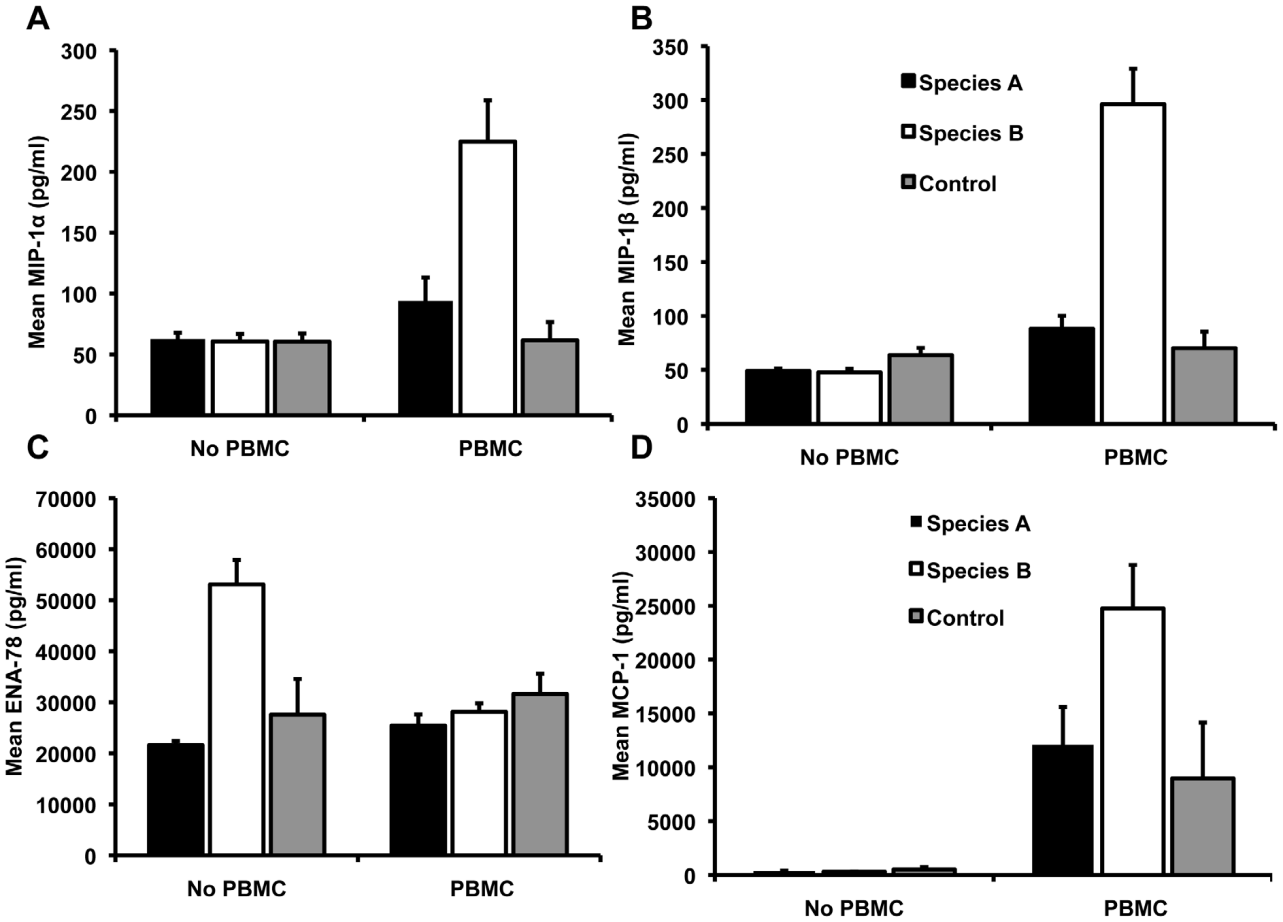
HRV species associated cytokine/chemokine production in calu-3 cells. Fig. 1A, 1B, 1C and 1D. Levels of cytokines/chemokines (pg/ml) in the supernatant of calu-3 cells infected with species A (HRV 25, HRV 31 and HRV 36) or species B (HRV 4, HRV 35 and HRV 48) HRVs and uninfected control cells with or without PBMCs. Data are mean from duplicate infection using three PBMC donors (n = 3).

Interestingly, species associated significant differences in responses (significant difference between values for all group A compared to values for all group B HRVs) were evident for MIP-1α, MIP-1β and MCP-1 after the addition of PBMCs and ENA-78 without PBMCs (Fig. 1A, 1B, 1C and 1D). For MIP-1α, MIP-1β and MCP-1, species B HRVs showed a significant HRV-associated response after the addition of PBMCs (p<0.001), species A HRVs did induce an HRV-associated response and, with the addition of PBMCs, levels for HRV species B were significantly higher than those for HRV species A (p<0.001). Without PBMCs, HRV species B expressed higher levels of ENA-78 compared to species A and controls (p<0.001). The addition of PBMCs significantly decreased ENA-78 expression in HRV species B (p<0.001), had no effect on ENA-78 expression for species A and mock infected control cells, and eliminated the difference in levels between HRV species A and B and mock infected cells (Fig. 1C).

Next, to determine if memory responses induced by prior HRV infection might contribute to responses we detected, we tested CBMCs from 3 infants. For CBMC experiments, we included the previously tested HRV 16 (species A) and HRV 14 (species B) viruses [26] with the 6 HRVs in the present study. Though the CBMCs did increase the magnitude of MCP-1 and INF-α, the increase was similar for infected and mock-infected cells and consequently did not change the pattern in HRV-associated responses seen without CBMCs (Table 2) with 3 exceptions. The addition of CBMCs to HRV 36 and HRV 35 did give an HRV-associated increase in IP-10 levels not seen with PBMCs (Table 2) and, though the addition of CBMCs to HRV 36- and 31-infected cells did not significantly change ENA 78 levels, it was associated with a lower level for mock-infected cells resulting in an HRV-associated responses, significantly different from mock infected cells, not seen with PBMCs. Finally, we compared the levels of cytokines/chemokines secreted with PBMCs to that with CBMCs exposed to HRV infected cells. Table 3 shows significant differences in up regulation or down regulation of certain cytokines and chemokines with PBMCs or CBMCs associated with different HRVs. MIP-1α and IFN-α levels were significantly higher (p<0.05) with some strains of HRV after PBMC exposure but not after CBMC exposure (Table 3). In contrast, ENA-78 levels with species B viruses were suppressed with addition of PBMCs but not with the addition of CBMCs. Consequently, ENA-78 levels were significantly lower (p<0.05) for species B virus infected cells with the addition of PBMCs compared to those with the addition of CBMCs (Table 3).

**Table 2:** HRV-associated responses in calu-3 cells with or without CBMCs.

CBMC	Virus	MIP-1α	MIP-1β	MCP-1	ENA-78	IP-10	IFN-α	HGF	IL-15
**No**	**HRV 25**	40*	44	654	35021	159	31	98	236*
	**HRV 31**	39	37	506	31285	151	23	92	125*
	**HRV 36**	38	35	439	33890	170	24	153	51
	**HRV 16**	37	35	533	41130*	304*	24	82	30
	**HRV 4**	38	34	533	59513*	149	24	87	49
	**HRV 35**	36	32	365	58043*	145	24	92	48
	**HRV 48**	38	34	351	53813*	447*	27	101	149*
	**HRV 14**	29	34	269	58555*	1158*	19	62	54
	**Control**	33	33	416	29125	153	41	62	48
**Yes**	**HRV 25**	56	75	3737	32616	435	157	101	169
	**HRV 31**	44	67	3113	35991*	137	119	92	107
	**HRV 36**	79	77	3456	31344*	474*	169	132	162
	**HRV 16**	64	67	4688	36628*	855*	193	92	70
	**HRV 4**	53	49	5837	58390*	361	191	131	206
	**HRV 35**	54	60	4146	56605*	404*	136	72	107
	**HRV 48**	69	65	2541	47299*	736*	158	82	170
	**HRV 14**	174	456	3675	57335*	644*	201	92	147
	**Control**	91	56	4002	21868	208	188	101	90

Shown are the median values of cytokines and chemokines (pg/ml) produced in response to various HRVs and uninfected control infections at 24 hours after the time inserts with CBMCs were added (n = 3). Significant differences in cytokine/chemokine levels for HRV infected cells when compared to uninfected controls were calculated using Wilcoxen rank sum tests. * indicates significantly different from control (p<0.05).

**Table 3:** Comparison of CBMC vs. PBMC.

Group	Virus	MIP-1α	MIP-1β	MCP-1	ENA-78	IP-10	IFN-α	HGF	IL-15
**PBMC**	**HRV 25**	76	116	10697	29776	127	229	158	216
	**HRV 31**	62	70	8978	23703	67	227*	113	147
	**HRV 36**	55	84	18180	20000*	64*	209	126	166
	**HRV 4**	181	254	22378*	29705*	53*	222	177	181
	**HRV 35**	184*	254	18614*	28132*	76*	242	132	144
	**HRV 48**	255*	340	19696*	23049*	434	245*	186	181
	**Control**	66	49	408	24423	86*	62	43	109
**CBMC**	**HRV 25**	56	75	3737	32616	435	157	101	169
	**HRV 31**	44	67	3113	35991	137	119	92	107
	**HRV 36**	79	77	3456	31344	474	169	132	162
	**HRV 4**	53	49	5837	58390	361	191	131	206
	**HRV 35**	54	60	4146	56605	404	136	72	107
	**HRV 48**	69	65	2541	47299	736	158	82	170
	**Control**	33	33	416	29125	153	41	62	48

Comparison of HRV-associated response in calu-3 cells with CBMCs and PBMCs. Shown are the median values of cytokines and chemokines (pg/ml) produced in response to various HRVs and uninfected control infections at 24 hours after the time inserts with PBMCs or CBMCs were added (n = 3). Significant differences in cytokine/chemokine levels for HRV infected cells comparing CBMCs and PBMCs were calculated using Wilcoxen rank sum tests. * indicates significantly different in values with CBMCs compared to those with PBMCs (p<0.05).

**Figure 2:**
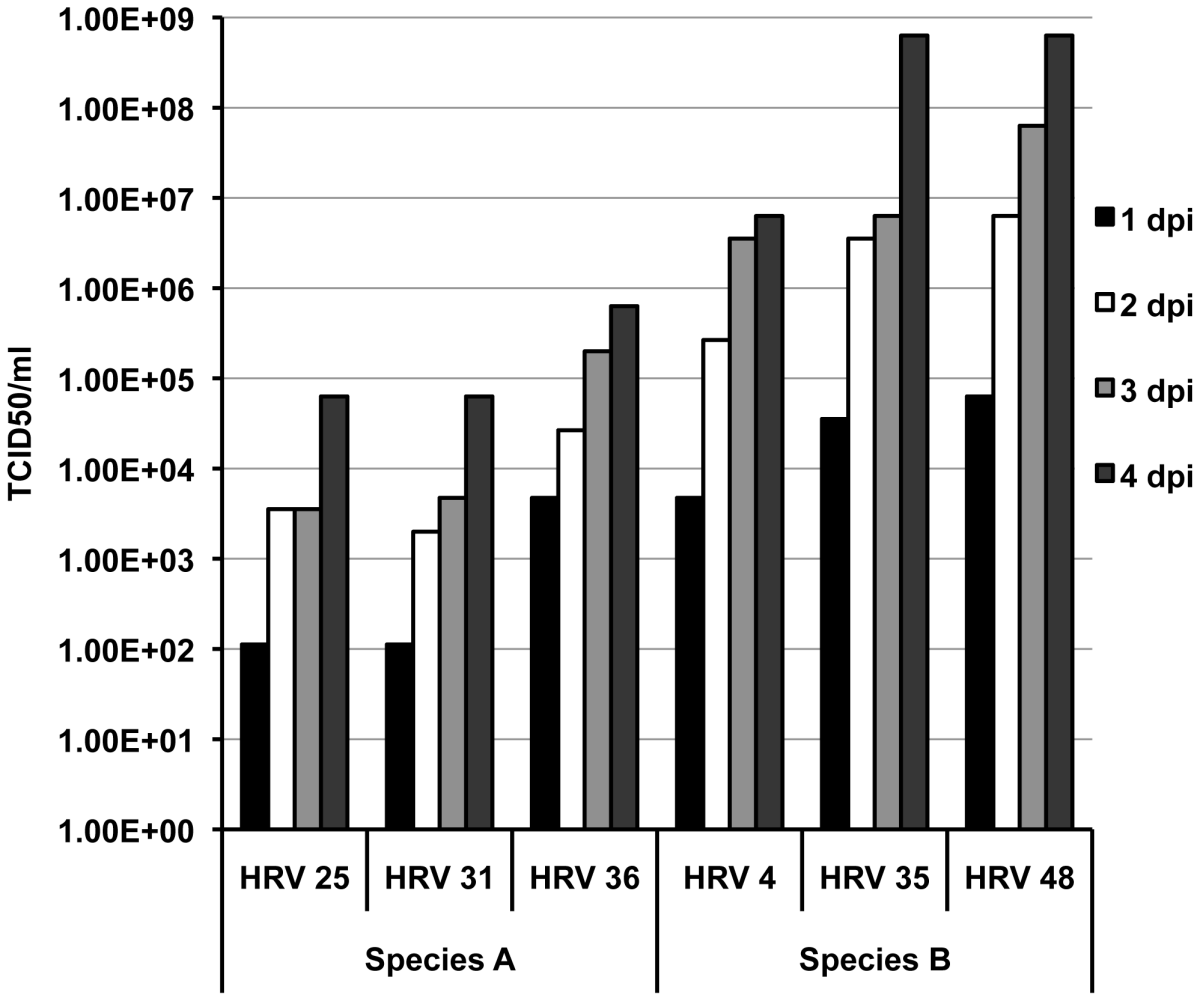
Time course of replication in HRV infected Calu-3 cells. Calu-3 cells were infected with serial dilutions of viruses in 8 replicates and infectious virus TCID_50_ in cell supernatant was determined in a microneutrlization assay and calculated using Reed Muench method.

We also determined the replication of the viruses using RT PCR method (Fig. 2). Different HRVs have different replication capabilities and it is possible that differences in virus replication caused some of the cytokine and chemokine differences we saw. As shown in Fig. 2, species B HRVs replicated better than species A HRVs.

## Discussion

Epidemiologic studies suggest the three HRV species, A, B and C, have differences in illness patterns. For example in some studies, species A and C HRVs have stronger associations with exacerbations of asthma and acute respiratory illness than species B HRVs [17], [18], [19], [20], [21], [22], [23], [24]. These epidemiologic observations and our recent finding [26] that HRV 14 (a group B virus) and HRV 16 (a group A virus) infection of calu-3 cells with PBMCs induce different cytokine and chemokine responses led us to look for strain and species differences in responses induced by *in vitro* HRV infection. We hypothesized that HRV strain or species differences in disease might be associated with and possibly explained by species differences in cytokine and chemokine responses detected by our *in vitro* model. Previously we and others have shown that a variety of cytokines and chemokines were induced by both HRV 14 and/or HRV 16 infection *in vitro* that include FGF-Basic, chemokines and cytokines like IL-6, IL-15, MCP-2, IP-10, MIP-1β, type I IFN-α, type III IFN-λ2 (IL-28A), and ENA-78 [26], [31], [32]. Several groups have also studied cytokine or chemokine responses during HRV infection in respiratory specimens from patients with and without asthma. Gern et al reported that inoculation of allergic individuals with HRV led to increases in cytokine levels in respiratory secretions including IL-8 and granulocyte colony stimulating factor (GCSF) that was associated with increased neutrophils in sputum [33]. Miller et al detected an association between increased levels of Type III IFN-λ and wheezing in asthmatic patients with HRV infection [34]. The cytokines and chemokines induced by HRV infection are associated with a range of functions including activation of immune cells that are implicated in allergic responses, cell proliferation, anti-viral activity, and pro-inflammatory and anti-inflammatory functions [35]. In the present study, we describe the cytokine and chemokine responses seen with 3 species A viruses, HRV 25, HRV 31 and HRV 36, and 3 species B viruses, HRV 4, HRV 35 and HRV 48, and differences in induction of cytokines and chemokines including some that are common to HRVs from one but not the other species.

Although we analyzed a variety of cytokines and chemokines that are associated with HRV infection, we detected only a few which are significantly higher than uninfected controls and associated with HRV infection. Most of the cytokines and chemokines in mock infected control wells showed similar levels to HRV infected cells. We found increases in IP-10, IL-15, IFN-α, HGF, MIP-1α, MIP-1β, and ENA-78 with some or all of the HRVs. For IL-15, IP-10, HGF and ENA-78 the increase occurred without PBMCs while the increase only was noted with PBMCs for IFN-α, MIP-1α, and MIP-1β. Increases for IFN-α and HGF were noted for all 6 viruses (excluding HRV 31 for HGF) while increases for MIP-1α, MIP-1β, MCP-1 and ENA-78 were only seen with the HRV species B. Findings from our earlier study support species differences in induction of MIP-1β and ENA-78 [26] but not for MIP-1α. In that study, the species B HRV, HRV 14, showed significant HRV-associated increases in MIP-1β and ENA-78 while the species A HRV, HRV 16, did not and neither showed a significant HRV-associated increase in MIP-1α. As in the present study, our earlier study showed HRV-associated increase in ENA-78 for the group B virus was only evident without PBMCs. PBMCs down regulated the ENA-78 response to levels similar to that induced by the group A HRV- and mock-infected A549 cells. ENA-78 has been reported to be induced by HRV infection of BEAS-2B cells and suggested to have a role in allergy and airway inflammation [36], [37]. A possible mechanism for the decrease in levels of some cytokine and chemokines after the addition of PBMCs is suggested by reports showing that PBMCs can induce receptor mediated consumption of cytokines or release mediators that regulates cytokine or chemokine degradation [38], [39]. In contrast to ENA-78, the increase in MIP-1α and MIP-1β for species B but not species A HRV was only present after the addition of PBMCs. Thus, the combined results from the earlier and the present study suggest species specific responses for MIP-1β and ENA-78.

Most of the *in vitro* studies on the response of human immune cells to HRV infection have used adult PBMCs. Since adults will have already been infected with multiple HRVs, it is possible that memory responses induced by these previous infections might have contributed to some of our findings. Since there is cross reactivity among serotypes [40], [41], [42], previous infections by the same or heterologous serotypes might lead to such memory responses. Our findings that CBMCs, which are likely HRV naïve, usually had less effect on HRV-associated responses suggest that differences in HRV memory might have contributed to some of the differences we saw. The difference between CBMC- and PBMC-associated responses might also result from differences in their ability to respond to HRV infection. The immune system of the fetus and young infant is immature, and therefore CBMCs may be inherently less able to respond to HRV infection. Weitzel et al., reported differences in the expression of microRNAs derived from adults and neonates that regulate the transcription of cytokine genes [43], [44]. We did note some differences in levels of cytokines and chemokines but, in most instances, changes were similar for HRV-infected and uninfected cells and, thus, the addition of CBMCs, unlike the addition of PBMCs, usually did not change the pattern of HRV-associated responses. Previously, lower levels of MCP-1 and IL-1Ra were associated with increased severity of HRV infection [45] and a new report showed that MCP-1 production by epithelial cells and macrophages contributes to HRV-induced airway hyper responsiveness and inflammation in a mouse model of allergic airways disease [46]. In our study, HRV infection did not lead to a significant increase in MCP-1 levels though we noticed that species B viruses induced higher levels than species A viruses. In addition, previously studied HRV 14 and HRV 16 utilize the same receptor, ICAM1. However, two of the species A viruses HRV 25 and 31 in this study use the minor LDLR allowing comparisons across species and receptor usage. We did not note any receptor-associated differences in responses. Differences in cytokine and chemokine production detected with this model of the host response to HRV infection system should provide a way to explore the effect of virus and host differences on HRV disease.

In conclusion, the results of this study show serotype differences in the cytokine and chemokine responses to infection with our *in vitro* model and some of these differences may be species A or B specific. The differences that we noticed between PBMCs and CBMCs emphasize the need to recognize the potential that memory responses and/or maturity of immune cells need to be considered in interpreting results. This model system of the host response to HRV infection applied to HRVs and PBMCs linked to different disease outcomes should help clarify viral and host factors that contribute to HRV disease.
